# Diversity and abundance of *Culicoides* on goat and cattle farms in the southern part of the Republic of Korea

**DOI:** 10.1051/parasite/2026005

**Published:** 2026-02-10

**Authors:** Seung Bak An, Jiseung Jeon, Jihun Ryu, Jong-Uk Jeong, In-Soon Roh, Kwang Shik Choi

**Affiliations:** 1 School of Life Sciences, BK21 FOUR KNU Creative BioResearch Group, Kyungpook National University Daegu 41566 Republic of Korea; 2 Department of Biology, College of Natural Sciences, Kyungpook National University Daegu 41566 Republic of Korea; 3 Vector-Borne Disease Laboratory, Foreign Animal Disease Division, Animal and Plant Quarantine Agency Gimcheon 39660 Republic of Korea

**Keywords:** Biting midges, *Culicoides*, Species diversity, Distribution, Unrecorded species

## Abstract

Biting midges of the genus *Culicoides* Latreille (Ceratopogonidae) pose a significant threat to veterinary health as vectors of over 60 viruses, most of which affect livestock. In this study, we used light traps to sample *Culicoides* populations on cattle and goat farms from May to October 2023 at 15 sites in Gyeongsangnam-do, Jeollanam-do, and Jeju Island, South Korea. Diversity and abundance were analysed based on the collection date, environmental conditions, and host species. A total of 124,055 individuals were collected, comprising 14 previously recorded and two newly recorded species: *C. asiana* and *C. palawanensis*. The dominant species was *C. arakawae*, which accounted for 80.60% of the total collected individuals, followed by *C. punctatus* (10.25%), and *C. tainanus* (3.36%), while the remaining 13 species constituted 5.80% of the collection. Total *Culicoides* abundance peaked in August (40.15%), driven largely by fluctuations in *C. arakawae* abundance, but the seasonal abundances of individual species varied. *Culicoides arakawae* and *C. punctatus* were dominant on the mainland, while *C. matsuzawai*, *C. lungchiensis*, and *C. tainanus* were dominant on Jeju Island. The dominant species on cattle farms were *C. arakawae* and *C. punctatus,* while *C. arakawae* dominated in collections from goat farms. The detection of two new species records suggests that the fauna of South Korea is still incompletely understood.

## Abstract (fr)

Les moucherons piqueurs du genre *Culicoides* Latreille (Ceratopogonidae) constituent une menace importante pour la santé animale, car ils sont vecteurs de plus de 60 virus, dont la plupart affectent le bétail. Dans cette étude, nous avons utilisé des pièges lumineux pour échantillonner les populations de *Culicoides* dans des élevages caprins et bovins, de mai à octobre 2023, sur 15 sites répartis dans les provinces de Gyeongsangnam-do, Jeollanam-do et sur l’île de Jeju. La diversité et l’abondance ont été analysées en fonction de la date de collecte, des conditions environnementales et de l’espèce hôte. Au total, 124 055 individus ont été collectés, appartenant à 14 espèces déjà répertoriées et à deux espèces nouvellement répertoriées : *C. asiana* et *C. palawanensis*. L’espèce dominante était *C. arakawae*, représentant 80,60 % du total des individus collectés, suivie de *C. punctatus* (10,25 %) et *C. tainanus* (3,36 %), tandis que les 13 autres espèces constituaient 5,80 % des collectes. L’abondance totale des *Culicoides* a atteint un pic en août (40,15 %), principalement en raison des fluctuations de l’abondance de *C. arakawae*, mais l’abondance saisonnière des différentes espèces variait. Les espèces *C. arakawae* et *C. punctatus* étaient dominantes sur le continent, tandis que *C. matsuzawai*, *C. lungchiensis* et *C. tainanus* l’étaient sur l’île de Jeju. Les espèces dominantes dans les élevages bovins étaient *C. arakawae* et *C. punctatus*, tandis que *C. arakawae* dominait dans les collections provenant des élevages caprins. La détection de deux espèces nouvellement répertoriées suggère que la faune de la Corée du Sud est encore mal connue.

## Introduction

The genus *Culicoides* Latreille is a group of small, haematophagous midges, typically measuring fewer than 3 mm in length. It belongs to the family Ceratopogonidae, along with three other haematophagous genera – *Austroconops* Wirth and Lee, *Leptoconops* (Skuse), and *Forcipomyia* Meigen – and many non-haematophagous genera [8]. Among these genera, *Culicoides* is the most extensively studied with 1,413 species reported, representing 33 subgenera and many species remaining unplaced into subgenera [11, 12].

*Culicoides* species feed on a wide range of vertebrates, with some serving as vectors for various viral, protozoan, and parasitic pathogens [43]. Species of *Culicoides* transmit over 60 viruses, including bluetongue virus (BTV), Schmallenberg virus (SBV), and African horse sickness virus (AHSV), which primarily affect the livestock industry and are considered significant in the veterinary field [16, 43, 44]. In the Republic of Korea (ROK), infection with the arboviruses Akabane virus (AKAV), Chuzan virus (CHUV), Aino virus (AINV), Bovine ephemeral fever virus (BEFV), and Ibaraki virus (IBAV) have been reported in either livestock or midges [2, 3, 31, 38, 48, 51]. Currently, 12 species of *Culicoides* associated with disease transmission are known to inhabit the ROK. Pathogens have been detected in Korean specimens of four species (*Culicoides arakawae* (Arakawa), *C. oxystoma* Kieffer, *C. punctatus* (Meigen), and *C. tainanus* Kieffer [47, 65]), while the remaining eight species (*C. actoni* Smith, *C. chiopterus* (Meigen), *C. circumscriptus* Kieffer, *C. jacobsoni* Macfie, *C. lungchiensis* Chen & Tsai, *C. obsoletus* (Meigen), *C. sumatrae* Macfie, and *C. pulicaris* (Linnaeus)) are associated with pathogen transmission in other countries [16, 19, 21, 43, 44, 45, 49, 64, 66].

Early studies on *Culicoides* diversity in the ROK were sporadic [1, 33, 56], until Cho and Chong [14] conducted the first comprehensive nationwide investigation. More recently, the diversity and abundance of *Culicoides* was investigated in Gyeonggi-do, Gyeongsang-do, and Jeolla-do, and on Jeju Island [26, 27, 28, 30, 65]. Basic morphological descriptions of 16 *Culicoides* species commonly found in the country were provided by Choi *et al.* [15], along with their distributions, while Bellis *et al.* [7] and Jeon *et al.* [23] each reported three previously unrecorded species; Kim *et al.* [28] and Lee *et al.* [35] each added one species. Currently, a total of 38 species are recorded in the National Species List of the ROK and cross-referenced with the species catalogued in Borkent and Dominiak [11] (Table 1). Additionally, *C. pulicaris* (Linnaeus), 1758 was suggested to be a misidentification of *C. punctatus* by Kim *et al.* [26] and Bellis *et al.* [7]. The list for the ROK appears to be low compared to that of its neighbour Japan, where 83 species have been reported [1, 34, 60]. Furthermore, previous Korean studies have predominantly concentrated on Gyeonggi-do, Gyeongsang-do, and Jeju Island, resulting in insufficient data for other regions.

**Table 1:** List of *Culicoides* species reported in the ROK.

Subgenus	Species	References
*Amossovia*	*C. dendrophilus* Amosova, 1957	[13^a^, 14, 30]
*Avaritia*	*C. actoni* Smith, 1929	[14^b^]
	*C. asiana* Bellis, 2015	This study
	*C. chiopterus* (Meigen, 1830)	[29]
	*C. jacobsoni* Macfie, 1934	[7]
	*C. obsoletus* (Meigen, 1818)	[1, 14, 29]
	*C. sinanoensis* Tokunaga, 1937	[14, 27, 29, 30]
	*C. tainanus* Kieffer, 1916	[14^c^, 26, 27, 28, 30, 47, 64]
*Beltranmyia*	*C. circumscriptus* Kieffer, 1918	[1, 14, 24, 26–28, 30]
	*C. dokdoensis* Lee and Bae, 2023	[35]
	*C. japonicus* Arnaud, 1956	[14, 27]
	*C. koreensis* Arnaud, 1956	[1, 14, 27]
	*C. pallidulus* Yu, 1991	[7, 27, 30]
	*C. sphagnumensis* Williams, 1955	[14^d^, 27]
	*C. toyamaruae* Arnaud, 1956	[14]
	*C. verbosus* Tokunaga, 1937	[23]
*Culicoides*	*C. dubius* Arnaud, 1956	[14]
	*C. lungchiensis* Chen and Tsai, 1962	[28]
	*C. nipponensis* Tokunaga, 1955	[1^e^, 14, 24, 26, 27, 30, 56, 65]
	*C. pulicaris* (Linnaeus, 1758)	[4^g^, 14^g^, 24^g^, 29^g^]
	*C. punctatus* (Meigen, 1804)	[26, 28, 30, 47, 65]
*Fastus*	*C. erairai* Kono and Takahasi, 1940	[14, 26, 27, 29, 30]
*Hoffmania*	*C. sumatrae* Macfie, 1934	[14^h^, 24^i^]
*Meijerehelea*	*C. arakawae* (Arakawa, 1910)	[1, 14, 24, 26–28, 30, 47, 65]
*Monoculicoides*	*C. homotomus* Kieffer, 1922	[14, 24, 26, 27, 30]
*Oecacta*	*C. miharai* Kinoshita, 1918	[14, 33]
	*C. morisitai* Tokunaga, 1940	[14^j^, 27, 30]
	*C. nasuensis* Kitaoka, 1984	[7, 30]
	*C. omogensis* Arnaud, 1956	[14]
	*C. pictimargo* Tokunaga and Shogaki, 1953	[1, 14, 27, 30]
	*C. saninensis* Tokunaga, 1956	[14]
*Remmia*	*C. oxystoma* Kieffer, 1910	[4^k^, 14^k^, 26, 47]
*Sensiculicoides*	*C. festivipennis* Kieffer, 1914	[14^l^, 27, 30, 56^l^]
	*C. kibunensis* Tokunaga, 1937	[1, 59, 14^m^, 26, 27, 28, 30, 56^n^]
*Trithecoides*	*C. matsuzawai* Tokunaga, 1950	[14, 28]
Unplaced clavipalpis group	*C. clavipalpis* Mukerji, 1931	[14, 30]
Unplaced ornatus group	*C. circumbasalis* Tokunaga, 1959	[23]
	*C. palawanensis* Delfinado, 1961	This study
Unplaced shermani group	*C. thurmanae* Witrh and Hubert, 1989	[23]
Unplaced	*C. longidens* Arnaud, 1956	[14, 30]

^a^ Reported as *C. reesi* Bullock & Akiyama; ^b^ Reported as *C. okumensis* Arnaud; ^c^ Reported as *C. sigaensis* Tokunaga; ^d^ Reported as *C. laciocola* Arnaud; ^e^ Misidentified as *C. peregrinus* Kieffer; ^g^ Suggested as misidentification of *C. punctatus* in Kim *et al.* [26] and Bellis *et al.* [7]; ^h^ Reported as *C. amamiensis ohmorii* Takahashi; ^i^ Reported as *C. amamiensis* Tokunaga; ^j^ Reported as *C. nagahanai* Tokunaga; ^k^ Misidentified as *C. schultzei* (Enderlein); ^l^ Reported as *C. odibilis* Austen; ^m^ Reported as *C. ponkikiri* Kono and Takahasi; ^n^ Misidentified as *C. odiatus* Austen.

With the continuous progression of global warming, the ROK is expected to gradually shift towards a subtropical climate [17]. This is expected to increase the activity, affect the habitat, and influence the overwintering of disease vectors such as *Culicoides* [50]. Thus, this study aimed to survey the diversity and abundance of *Culicoides* in the southern part of the ROK, where the effects of climate change are expected to be the greatest [32].

## Materials and methods

**Figure 1:**
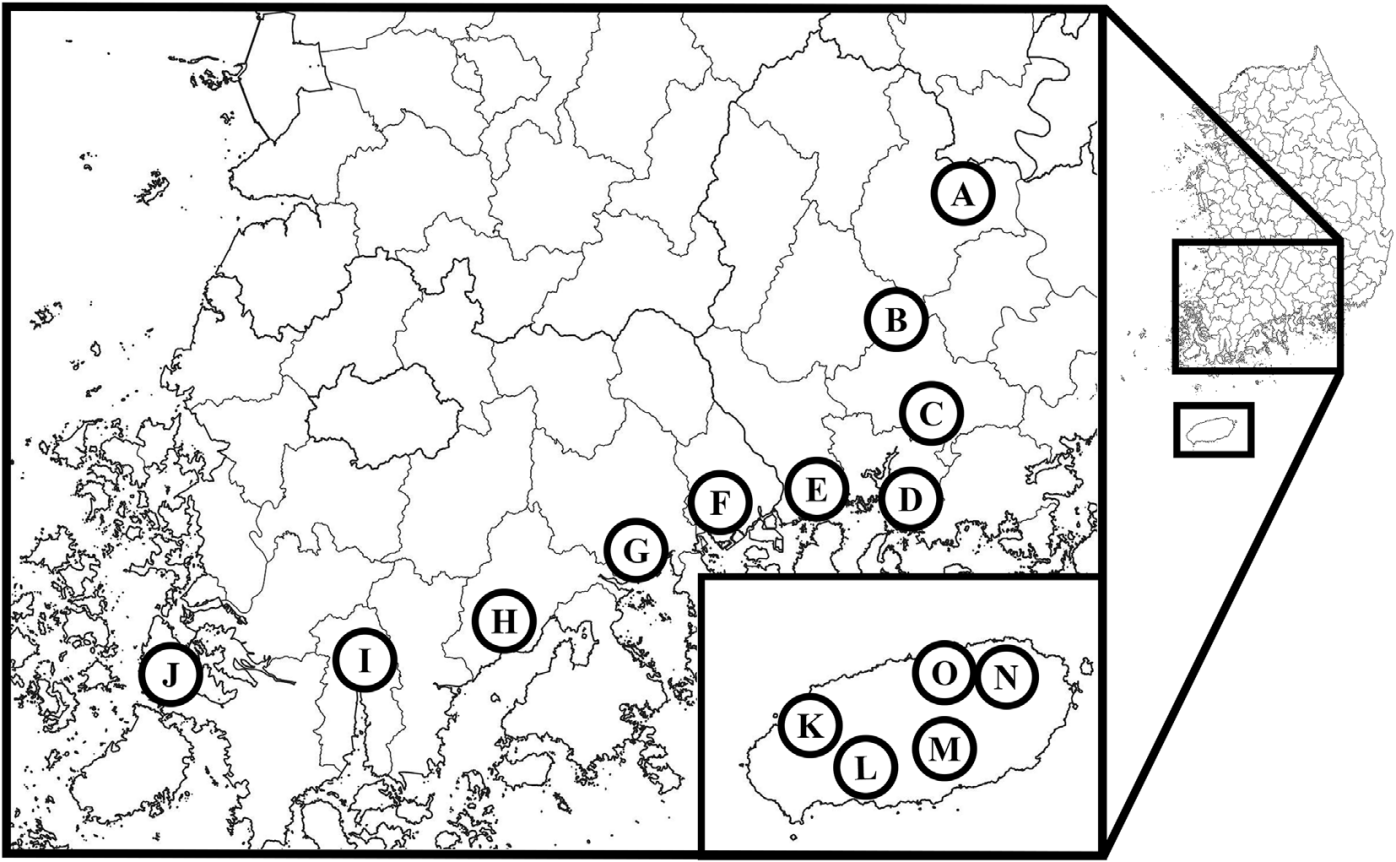
Collection sites for the study. A. Hapcheon, B. Sancheong, C. Jinju, D. Sacheon, E. Hadong, F. Gwangyang, G. Suncheon, H. Boseong, I. Gangjin, J. Haenam, K. Jeju1, L. Jeju2, M. Jeju3, N. Jeju4. O. Jeju5; Cow shed (A, B, C, F, I, J, K, L, N, O), Goat shed (D, E, G, H, M).

A total of 15 collection sites were selected in the southern regions of the ROK: five in Gyeongsangnam-do (three cattle sheds and two goat sheds), five in Jeollanam-do (three cattle sheds and two goat sheds), and five on Jeju Island (four cattle sheds and one goat shed) (Table 2, Fig. 1). Collections were made over a single night every two weeks from May to September, and once in October 2023. Collection dates on the mainland (Gyeongsangnam-do and Jeollanam-do) were the same for all sites, while collection dates for Jeju Island sites differed from those of the mainland by a few days. Blacklight traps equipped with removable fine mesh bags for the collection of *Culicoides* species, and a CO_2_ release agent as an auxiliary attractant (model: “ultra trap”, BT Global Co., Ltd., Seongnam, Republic of Korea) were used for collections. Traps were installed inside sheds near host species, and fixed over 1.5 m from land during collection periods. Traps were retrieved one day after installation and transferred to the laboratory. All collected samples were stored in a −70 °C freezer until species identification.

**Table 2:** Information on *Culicoides* species collection sites.

Province		Collection sites	Host present	Number	Latitude and longitude
Gyeongsangnam-do	A	Hapcheon	Cattle	800	35°31′59″N 128°15′43″E
	B	Sancheong	Cattle	300	35°19′30″N 127°59′55″E
	C	Jinju	Cattle	150	35°08′01″N 128°09′15″E
	D	Sacheon	Goat	200	35°05′01″N 128°01′27″E
	E	Hadong	Goat	40–100	35°00′56″N 127°51′04″E
Jeollanam-do	F	Gwangyang	Cattle	100	35°00′16″N 127°36′23″E
	G	Suncheon	Goat	100	34°52′34″N 127°26′19″E
	H	Boseong	Goat	150	34°46′01″N 127°06′27″E
	I	Gangjin	Cattle	300	34°42′45″N 126°46′28″E
	J	Haenam	Cattle	100	34°38′15″N 126°19′55″E
Jeju Island	K	Jeju1	Cattle	200	33°21′54″N 126°20′17″E
	L	Jeju2	Cattle	70	33°17′07″N 126°28′09″E
	M	Jeju3	Goat	1500–2000	33°19′03″N 126°39′35″E
	N	Jeju4	Cattle	110	33°27′56″N 126°47′16″E
	O	Jeju5	Cattle	100	33°28′29″N 126°39′32″E

Species occurrences were sorted according to the collection region and date. The Statistical Package for the Social Sciences (SPSS) [53] was used to conduct Pearson and Spearman correlation analyses assessing the seasonal occurrence in relation to climate parameters, including temperature and precipitation, obtained from the Korea Meteorological Administration (https://data.kma.go.kr/cmmn/main.do) for the collection locations. Climate parameters used for analyses were based on a mean of the two-week period before each collection.

Preliminary identification was conducted using a stereoscopic microscope (SZ61, Olympus Corporation, Tokyo, Japan). Genus identification was based on morphological keys from Borkent [10], McAlpine *et al.* [41], and Swanson [52]. Species identification was based on several previous studies [1, 6, 7, 8, 15, 18, 34, 55, 56, 57, 60, 61, 67]. Before DNA extraction, non-destructive tissue digestion was performed. Specimens were digested at 55 °C for 2 h in 350 μL of DLD buffer, 3.5 μL of 2-mercaptoethanol, and 20 μL of proteinase (Invirustech, Gwangju, ROK). Following digestion, specimens were stored in 70% ethanol. Later, cadavers were temporarily placed onto glass slides with 70% ethanol to measure the head, wings, and abdomen using a camera (BUC5H-2000C, Beijing Bestscope Technology Co., Ltd., Beijing, PR China) connected to an optical microscope (CX43, Olympus). For the head, the contiguous or separated compound eyes, presence of pubescence between the ommatidia (facets), the shape of the third palpal segment, presence and shape of the sensory pit, palpal ratio (PR), distribution of sensilla coeloconica (SCo) on the flagellomeres of the antennae, antennal ratio (AR), and the length of the proboscis/head height (P/H) ratio were measured. The length and width of the wings were examined alongside the costal ratio (CR), and wing patterns were characterised. The position and presence of pale bands on the femora and tibiae, the number of hind tibial combs, and the order of the longest comb from the spur were measured for the legs. In the female genitalia, the number, shape, and size of the spermathecae were measured, and the presence and size of the sclerotised neck, rudimentary spermatheca, and sclerotised ring were measured. After completing the measurements, all samples were preserved at –20 °C in a freezer, in 70% ethanol.

To confirm the identification of unrecorded species, the mitochondrial cytochrome oxidase subunit 1 (COI) region was sequenced, and a phylogenetic analysis was performed. First, polymerase chain reactions (PCRs) were performed using the BC1culicFm and JerR2m primer pair [5]. The thermocycler protocol included an initial denaturation step at 94 °C for 2 min; 40 cycles of denaturation at 94 °C for 30 s, annealing at 48 °C for 30 s, and extension at 72 °C for 1 min; and a final 5-minute elongation step at 72 °C. Amplified PCR products were then submitted to Macrogen (Daejeon, ROK) for Sanger sequencing, obtaining bidirectional sequences using the primers M13F and M13R-pUC, the sequences of which are included in the sequences of BC1culicFm and JerR2m. The acquired sequences were aligned using BioEdit v.7.2.6.1 [22], registered in GenBank (accession numbers PQ643289–PQ643290 for *C. asiana* Bellis; PQ643291 and PV111011–PV111017 for *C. palawanensis* Delfinado), and then the sequences of each species were compared to those in GenBank using BLAST. Phylogenetic trees were produced using the neighbour-joining (NJ) method and pairwise distance analyses were performed for the unrecorded species using MEGA 11 [54]. Publicly available GenBank-registered sequences related to the two unrecorded species, *C. asiana* (MW496167, MW496170, MW496171, KJ162955, KJ162956, KT352310, KT352321, and KT352360) and *C. palawanensis* (KY441765, KY441793, KY441798, KY441807, KY441809, and ON002365) were compared to those of the collected samples, and *Alluaudomyia quadripunctata* (KT278187) was applied as an outgroup in both phylogenetic analyses.

## Results

**Figure 2:**
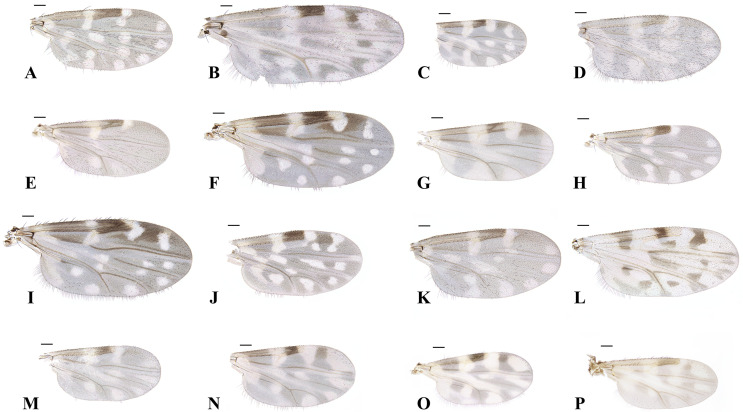
Wings of 16 collected *Culicoides* species including 2 newly recorded species (O and P): (A) *C. arakawae*, (B) *C. homotomus*, (C) *C. jacobsoni,* (D) *C. japonicus*, (E) *C. kibunensis*, (F) *C. lungchiensis*, (G) *C. matsuzawai*, (H) *C. morisitai*, (I) *C. nipponensis*, (J) *C. oxystoma*, (K) *C. pictimargo*, (L) *C. punctatus*, (M) *C. sinanoensis*, (N) *C. tainanus*, (O) *C. asiana*, (P) *C. palawanensis*. Scale bars = 0.1 mm.

A total of 124,055 *Culicoides* samples comprising 14 known domestic species and two newly recorded species: *C. asiana* and *C. palawanensis* were collected (Table 3, Fig. 2). The predominant species was *C. arakawae* (80.60%, 99,985), followed by *C. punctatus* (10.25%, 12,712), *C. tainanus* (3.36%, 4,167), *C. matsuzawai* (2.79%, 3,465) and *C. oxystoma* (1.30%, 1,617). The remaining 11 species, 2,108 individuals collectively, accounted for 1.70% of the total collection.

**Table 3:** Seasonal distribution of 16 *Culicoides* species collected in southern Korea, 2023.

	Month											
	MAY		JUN		JUL		AUG		SEP		OCT	Total (%^*2^)
Species	1st (%^*^)	2nd (%^*^)	1st (%^*^)	2nd (%^*^)	1st (%^*^)	2nd (%^*^)	1st (%^*^)	2nd (%^*^)	1st (%^*^)	2nd (%^*^)	1st (%^*^)	
*C. arakawae*	86 (0.09)	1,671 (1.67)	10,103 (10.1)	4,462 (4.46)	1,432 (1.43)	18,766 (18.77)	17,007 (17.01)	30,445 (30.45)	10,915 (10.92)	4,738 (4.74)	360 (0.36)	99,985 (80.60)
*C. punctatus*	157 (1.24)	1,187 (9.34)	4,223 (33.22)	270 (2.12)	868 (6.83)	2,936 (23.1)	931 (7.32)	518 (4.07)	577 (4.54)	524 (4.12)	521 (4.1)	12,712 (10.25)
*C. tainanus*	45 (1.08)	829 (19.89)	888 (21.31)	100 (2.4)	163 (3.91)	1,098 (26.35)	131 (3.14)	37 (0.89)	322 (7.73)	388 (9.31)	166 (3.98)	4,167 (3.36)
*C. matsuzawai*	2 (0.06)	7 (0.2)	47 (1.36)	2 (0.06)	4 (0.12)	2,911 (84.01)	37 (1.07)	85 (2.45)	234 (6.75)	136 (3.92)	0	3,465 (2.79)
*C. oxystoma*	0	2 (0.12)	23 (1.42)	12 (0.74)	14 (0.87)	430 (26.59)	241 (14.9)	87 (5.38)	427 (26.41)	356 (22.02)	25 (1.55)	1,617 (1.3)
*C. nipponensis*	0	0	18 (2.36)	14 (1.84)	41 (5.38)	283 (37.14)	2 (0.26)	44 (5.77)	146 (19.16)	214 (28.08)	0	762 (0.61)
*C. lungchiensis*	1 (0.14)	4 (0.55)	5 (0.69)	6 (0.83)	5 (0.69)	15 (2.07)	49 (6.75)	29 (3.99)	290 (39.94)	318 (43.8)	4 (0.55)	726 (0.59)
*C. morisitai*	20 (7.35)	3 (1.1)	10 (3.68)	16 (5.88)	2 (0.74)	3 (1.1)	34 (12.5)	100 (36.76)	77 (28.31)	7 (2.57)	0	272 (0.22)
*C. japonicus*	2 (1.16)	11 (6.4)	137 (79.65)	3 (1.74)	7 (4.07)	0	1 (0.58)	0	0	9 (5.23)	2 (1.16)	172 (0.14)
*C. palawanensis*	0	0	62 (75.61)	0	0	0	0	7 (8.54)	0	13 (15.85)	0	82 (0.07)
*C. asiana*	0	0	0	0	0	0	1 (2.17)	13 (28.26)	32 (69.57)	0	0	46 (0.04)
*C. kibunensis*	1 (4.35)	1 (4.35)	6 (26.09)	1 (4.35)	0	1 (4.35)	5 (21.74)	1 (4.35)	2 (8.7)	5 (21.74)	0	23 (0.02)
*C. homotomus*	3 (30)	0	2 (20)	2 (20)	0	1 (10)	0	2 (20)	0	0	0	10 (0.01)
*C. jacobsoni*	0	0	0	0	0	1 (14.29)	0	0	3 (42.86)	2 (28.57)	1 (14.29)	7 (0.01)
*C. sinanoensis*	0	0	1 (20)	0	3 (60)	1 (20)	0	0	0	0	0	5 (<0.01)
*C. pictimargo*	0	0	0	0	0	0	0	3 (75)	0	0	1 (25)	4 (<0.01)
Total (%^*3^)	317 (0.26)	3,715 (2.99)	15,525 (12.51)	4,888 (3.94)	2,539 (2.05)	26,446 (21.32)	18,439 (14.86)	31,371 (25.29)	13,025 (10.50)	6,710 (5.41)	1,080 (0.87)	124,055

* Number of *Culicoides* species in a single collection / Total number of each *Culicoides* species (%).

*2 Total number of each *Culicoides* species / Total number of collections (%).

*3 Total number of *Culicoides* species in a single collection / Total number of collections.

Regarding seasonal distribution, *Culicoides* individuals were most abundant in late August, when 25.29% (31,371) of the total collection was acquired, but this high proportion was mainly comprised of *C. arakawae* (30,445) (Table 3). Temporal distribution patterns differed among *Culicoides* species. The predominant species, *C. arakawae* was mainly collected during late July and early September, exhibiting its highest abundance in late August (30.45%, 30,445). *Culicoides punctatus* exhibited two peaks in early June (33.22%, 4,223) and late July (23.10%, 2,936), while *C. tainanus* was concentrated in late May (19.89%, 829), early June (21.31%, 888), and late July (26.35%, 1,098). *Culicoides matsuzawai* was mainly found in the late July (84.01%, 2,911) collection, and *C. oxystoma* was primarily collected in late July (26.59%, 430) and in both collection periods in September (26.41%, 427; 22.02%, 356).

**Figure 3:**
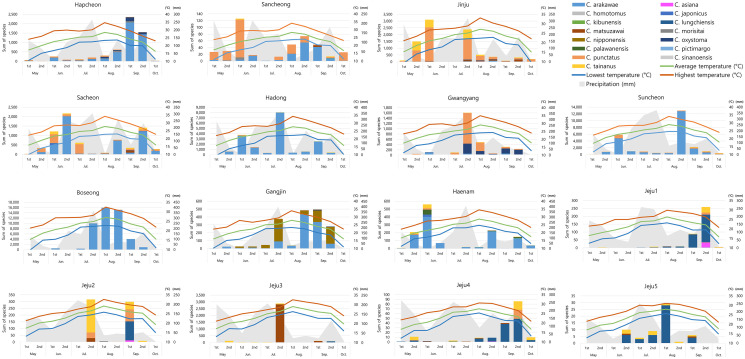
Seasonal distribution of *Culicoides* species at collection sites with climate parameters.

Geographic occurrence patterns also differed among species. *Culicoides arakawae* was collected from 12 locations, excluding Jeju2, Jeju4, and Jeju5, and was more abundant in goat sheds than in cattle sheds (cattle:goat = 7,222:92,763) (Tables 4 and 5, Fig. 3). Individual collection sites in which *C. arakawae* represented high proportions included cow sheds in Hapcheon (80.62%, 4,501), Sancheong (36.07%, 154), Gangjin (55.65%, 1,004), and Haenam (81.98%, 1,078), and goat sheds in Sacheon (67.47%, 4,933), Hadong (97.31%, 17,734), Suncheon (88.49%, 23,179), and Boseong (99.70%, 46,881) (Table 4). In contrast, *C. punctatus* was collected from 14 sites, excluding only Jeju5, and was more abundant in cow sheds (cattle:goat = 8,286:4,426). Sites at which *C. punctatus* represented high proportions included cow sheds in Sancheong (59.02%, 252), Jinju (66.22%, 5,671), Gwangyang (57.68%, 1,761), and Jeju2 (21.36%, 135), and considerable numbers were collected in goat sheds in Sacheon (22.01%, 1,609) and Suncheon (9.39%, 2,460). *Culicoides tainanus* was collected at all sites, representing high proportions in cow sheds at Jinju (25.04%, 2,144), Jeju2 (48.89%, 309), and Jeju4 (19.77%, 34), *C. matsuzawai* was collected at seven sites but was dominant only at Jeju3 (87.56%, 2,901), and *C. oxystoma* was collected from seven locations but exhibited a notable proportion only at Gwangyang (29.84%, 911). Among other species collected at relatively high abundance at one or more sites, *C. nipponensis* Tokunaga was exclusively collected at Gangjin (42.24%, 762), and *C. lungchiensis* was dominant at Jeju1 (72.92%, 280), Jeju2 (23.42%, 148), Jeju4 (65.12%, 112), and Jeju5 (79.66%, 47). For the newly recorded species, *C. asiana* was collected at Jeju1 (32), Jeju2 (13), and Jeju4 (1), while *C. palawanensis* was exclusively collected in Haenam (82).

**Table 4:** Total number (%^*^) of *Culicoides* species collected at 15 sites in southern Korea, 2023.

	Location															
Species	Hapcheon	Sancheong	Jinju	Gwangyang	Gangjin	Haenam	Jeju1	Jeju2	Jeju4	Jeju5	Sacheon	Hadong	Suncheon	Boseong	Jeju3	Total
*C. arakawae*	4501 (80.62)	154 (36.07)	195 (2.28)	287 (9.4)	1004 (55.65)	1078 (81.98)	3 (0.78)	0	0	0	4933 (67.47)	17734 (97.31)	23179 (88.49)	46881 (99.7)	36 (1.09)	99,985
*C. punctatus*	385 (6.9)	252 (59.02)	5671 (66.22)	1761 (57.68)	8 (0.44)	35 (2.66)	19 (4.95)	135 (21.36)	20 (11.63)	0	1609 (22.01)	231 (1.27)	2460 (9.39)	117 (0.25)	9 (0.27)	12,712
*C. tainanus*	21 (0.38)	5 (1.17)	2144 (25.04)	52 (1.7)	7 (0.39)	82 (6.24)	49 (12.76)	309 (48.89)	34 (19.77)	10 (16.95)	466 (6.37)	256 (1.4)	442 (1.69)	17 (0.04)	273 (8.24)	4,167
*C. matsuzawai*	0	5 (1.17)	415 (4.85)	0	0	0	0	27 (4.27)	5 (2.91)	2 (3.39)	110 (1.5)	0	0	0	2901 (87.56)	3,465
*C. oxystoma*	462 (8.28)	0	124 (1.45)	911 (29.84)	18 (1)	0	0	0	0	0	23 (0.31)	3 (0.02)	76 (0.29)	0	0	1,617
*C. nipponensis*	0	0	0	0	762 (42.24)	0	0	0	0	0	0	0	0	0	0	762
*C. lungchiensis*	1 (0.02)	0	2 (0.02)	32 (1.05)	0	1 (0.08)	280 (72.92)	148 (23.42)	112 (65.12)	47 (79.66)	5 (0.07)	0	4 (0.02)	0	94 (2.84)	726
*C. morisitai*	201 (3.6)	10 (2.34)	8 (0.09)	7 (0.23)	0	0	0	0	0	0	34 (0.47)	0	12 (0.05)	0	0	272
*C. japonicus*	1 (0.02)	0	2 (0.02)	0	0	30 (2.28)	0	0	0	0	126 (1.72)	0	13 (0.05)	0	0	172
*C. palawanensis*	0	0	0	0	0	82 (6.24)	0	0	0	0	0	0	0	0	0	82
*C. asiana*	0	0	0	0	0	0	32 (8.33)	13 (2.06)	1 (0.58)	0	0	0	0	0	0	46
*C. kibunensis*	3 (0.05)	1 (0.23)	2 (0.02)	1 (0.03)	1 (0.06)	7 (0.53)	0	0	0	0	5 (0.07)	0	1 (<0.01)	2 (<0.01)	0	23
*C. homotomus*	4 (0.07)	0	1 (0.01)	0	4 (0.22)	0	0	0	0	0	0	0	0	1 (<0.01)	0	10
*C. jacobsoni*	0	0	0	1 (0.03)	0	0	1 (0.26)	0	0	0	0	0	5 (0.02)	0	0	7
*C. sinanoensis*	4 (0.07)	0	0	1 (0.03)	0	0	0	0	0	0	0	0	0	0	0	5
*C. pictimargo*	0	0	0	0	0	0	0	0	0	0	0	1 (0.01)	1 (<0.01)	2 (<0.01)	0	4
Total	5,583	427	8,564	3,053	1,804	1,315	384	632	172	59	7,311	18,225	26,193	47,020	3,313	124,055

* Number of *Culicoides* species at collection site/Total number of *Culicoides* species at collection site.

**Table 5:** Comparison of *Culicoides* abundances by farm type and environment.

Species	Environment				Farm type			
	Mainland (Cow/Goat)	(%)*	Island (Cow/Goat)	(%)*^2^	Cow (Land/Island)	(%)*^3^	Goat (Land/Island)	(%)*^4^
*C. arakawae*	99,946 (7,219/92,727)	83.64	39 (3/36)	0.86	7,222 (7,219/3)	32.84	92,763 (92,727/36)	90.89
*C. punctatus*	12,529 (8,112/4,417)	10.49	183 (174/9)	4.01	8,286 (8,112/174)	37.68	4,426 (4,417/9)	4.34
*C. tainanus*	3,492 (2,713/1,181)	2.92	675 (402/273)	14.80	2,713 (2,311/402)	12.34	1,454 (1,181/273)	1.42
*C. matsuzawai*	530 (420/110)	0.44	2,935 (34/2,901)	64.36	454 (420/34)	2.06	3,011 (110/2,901)	2.95
*C. oxystoma*	1,617 (1,515/102)	1.35	0	0	1,515 (1,515/0)	6.89	102 (102/0)	0.10
*C. nipponensis*	762 (762/0)	0.64	0	0	762 (762/0)	3.46	0	0
*C. lungchiensis*	45 (36/9)	0.04	681 (587/94)	14.93	623 (36/587)	2.83	103 (9/94)	0.10
*C. morisitai*	272 (226/46)	0.23	0	0	226 (226/0)	1.03	46 (46/0)	0.05
*C. japonicus*	172 (33/139)	0.14	0	0	33 (33/0)	0.15	139 (139/0)	0.14
*C. palawanensis*	82 (82/0)	0.07	0	0	82 (82/0)	0.37	0	0
*C. asiana*	0	0	46 (46/0)	1.01	46 (0/46)	0.21	0	0
*C. kibunensis*	23 (15/8)	0.02	0	0	15 (15/8)	0.07	8 (8/0)	<0.01
*C. homotomus*	10 (9/1)	<0.01	0	0	9 (9/0)	0.04	1 (1/0)	<0.01
*C. jacobsoni*	6 (1/5)	<0.01	1 (1/0)	0.02	2 (1/1)	<0.01	5 (5/0)	<0.01
*C. sinanoensis*	5 (5/0)	<0.01	0	0	5 (5/0)	0.02	0	0
*C. pictimargo*	4 (0/4)	<0.01	0	0	0	0	4 (4/0)	<0.01
Total	119,495 (20,746/98,749)		4,560 (1,247/3,313)		21,993 (20,746/1,247)		102,062 (98,749/3,313)	

* (Number of *Culicoides* species inland/Total number of *Culicoides* species inland).

*2 (Number of *Culicoides* species on the island/Total number of *Culicoides* species on the island).

*3 (Number of *Culicoides* species in cattle/Total number of *Culicoides* species in cattle).

*4 (Number of *Culicoides* species in goats/Total number of *Culicoides* species in goats).

The species compositions of the *Culicoides* collected at each of the 15 collection sites was compared between environments (mainland *vs* island) and farm types (cattle *vs* goats) (Table 5). The ten collection sites located in Gyeongsangnam-do and Jeollanam-do were classified as mainland environments, while the five sites on Jeju Island were classified as island environments. In the mainland environment, a total of 119,495 individuals, representing 15 *Culicoides* species, were collected, whereas in the island environment, a total of 4,560 individuals from 7 *Culicoides* species were collected. This indicates comparatively lower species richness and diversity on the island. In mainland environments, the dominant species were *C. arakawae* (83.64%) and *C. punctatus* (10.49%). In contrast, the island collections exhibited markedly different compositions, with *C. matsuzawai* (64.36%), *C. lungchiensis* (14.93%), and *C. tainanus* (14.80%) being predominant.

To assess species compositions at the two farm types, the collections from the 10 cattle sheds and were compared to those from the five goat sheds. A total of 21,993 individuals, representing 15 *Culicoides* species, were collected from cattle sheds, while 102,062 individuals, comprising 12 *Culicoides* species were collected from goat sheds. In cattle sheds, *C. punctatus* (37.68%), *C. arakawae* (32.84%), and *C. tainanus* (12.34%) were collected in high proportions, while *C. arakawae* (90.89%) was dominant in goat sheds. Among the 16 collected species, 11 were identified at both types of farm, with four species (*C. nipponensis*, *C. sinanoensis*, *C. asiana*, and *C. palawanensis*) exclusively found in cattle sheds, and *C. pictimargo* restricted to goat sheds.

**Figure 4:**
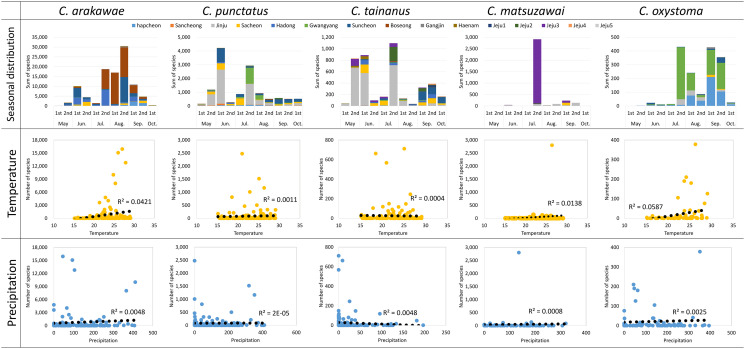
Seasonal distribution and scatter plot of five predominant species in relation to temperature and precipitation.

Correlation analyses were conducted for the five dominant species, *C. arakawae*, *C. punctatus*, *C. tainanus*, *C. matsuzawai*, *and C. oxystoma*, to examine correlations between occurrence and temperature and precipitation (Table 6, Fig. 4). *Culicoides arakawae* (*r* = 0.205, *p* = 0.018; *Rs* = 0.303, *p* < 0.001) and *C. oxystoma* (*r* = 0.242, *p* = 0.034; *Rs* = 0.382, *p* < 0.001) exhibited weak Pearson and Spearman correlation coefficients (*r* and *Rs*, respectively) in correlation with temperature. Additionally, *C. punctatus*, *C. tainanus*, and *C. matsuzawai* showed no statistically significant correlation with temperature; no species demonstrated a statistically significant correlation with precipitation.

**Table 6:** Correlation analysis about five predominant *Culicoides* species with temperature and precipitation.

			*C. arakawae*	*C. punctatus*	*C. tainanus*	*C. oxystoma*	*C. matsuzawai*
Pearson	Average temperature	*R*	0.205	0.034	−0.019	0.242	0.118
		*p*-value	0.018	0.679	0.810	0.034	0.309
		*N*	132	154	165	77	77
	Precipitation	*R*	0.069	0.004	−0.069	0.05	0.028
		*p*-value	0.43	0.959	0.379	0.669	0.812
		*N*	132	154	165	77	77
Spearman’s Rho	Average temperature	*R*	0.303	0.128	0.072	0.382	0.171
		*p*-value	<.001	0.114	0.359	<.001	0.138
		*N*	132	154	165	77	77
	Precipitation	*R*	0.018	−0.022	−0.091	−0.081	−0.019
		*p*-value	0.839	0.784	0.244	0.486	0.871
		*N*	132	154	165	77	77

### Taxonomy of unrecorded *Culicoides* species

#### Genus *Culicoides* Latreille, 1809: 251

Type species: *Culicoides punctatus* Latreille (= *Ceratopogon punctatus* Meigen), by monotypy.

#### Subgenus *Avaritia* Fox 1955: 218

Type species: *Ceratopogon obsoletus* Meigen, by original designation.

#### *Culicoides asiana* Bellis, 2015: 29 (formerly *Culicoides asiatica* Bellis, in Bellis et al. 2014: 407)

Non-type material examined: ROK, Jeju-do, 33°21′54″N 126°20′17″E, 23.09.27 (three females), accession number PQ643289; 33°17′07″N, 126°28′09″E, 23.09.16 (two females), accession number PQ643290; 33°27′56″N 126°47′16″E 23.08.22 (1 female).

Diagnosis: According to Bellis *et al.* [6] (as *C. asiatica*), this species is the only one in the Imicola complex that exhibits two specific characteristics: a pale spot at the apex of the m1 cell of the wing that extends distally but does not narrow or reach the M2 vein, and a dark spot on the base of the costa that is significantly longer than the dark spot on the stigma.

**Figure 5:**
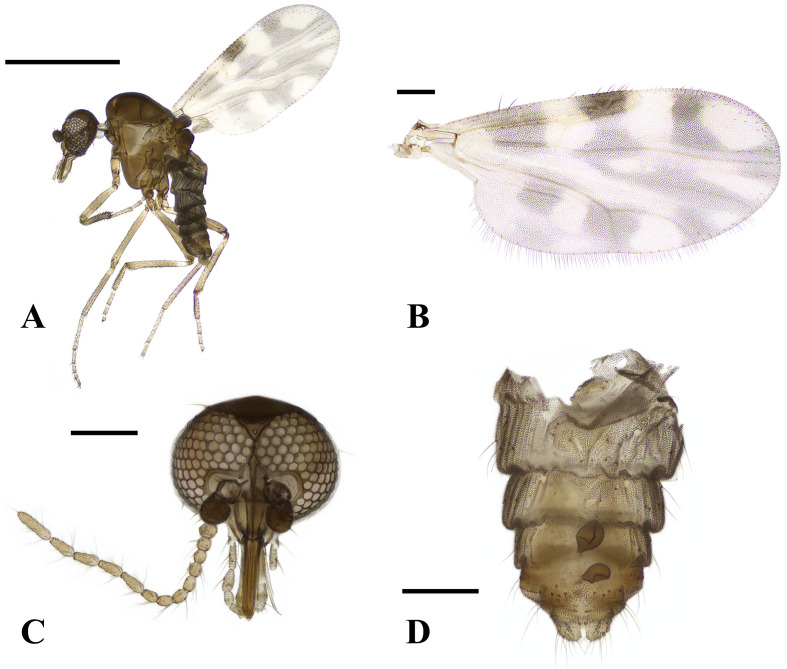
*Culicoides asiana* (A) lateral view, (B) wing, (C) head, (D) abdomen. Scale bars = 0.5 mm [A]; 0.1 mm [B, C, and D]).

Female (Figs. 2O and 5).

Body length 1.1 mm (1.01–1.25, *n* = 6). Head: Compound eyes contiguous, 26 μm or 2 facets, bare. Antennae with SCo on segments 1, 9–13; AR 1.26. Third palpal segment slightly swollen, with a shallow round sensory pit present. PR 2.2 (2.02–2.50, *n* = 5). P/H 0.71 (0.63–0.79, *n* = 5). Wings: 0.81 mm in length (0.77–0.88, *n* = 6) and 0.40 mm in width (0.38–0.44), CR 0.56 (0.54–0.58). Wing pattern matches those of previous records, as shown in Figure 2O. Abdomen: Two spermathecae present, oval-shaped and unequal in size, with developed neck. Rudimentary spermatheca and sclerotised ring present. Larger spermatheca 42 (39–46, *n* = 6) × 35 (32–41) μm, smaller one 33 (28–37) × 30 (26–36) μm, rudimentary spermatheca 12 (10–15) × 5 (4–5) μm, and sclerotised ring 8 (6–9) × 4 (3–5) μm.

Distribution: Japan, China, Taiwan, Malaysia, Indonesia, Thailand, Bangladesh, East Timor, Vietnam, Laos, and the ROK.

Remarks: *Culicoides asiana* is similar to the domestic species *C. tainanus*; the anal corner of *C. tainanus*’s wing is dark, and the pale spots in cells m1 and m2 are connected. In contrast, in *C. asiana*, the anal corner is pale, and the basal pale spot in cell m1 is distinctly separated from the spot in cell m2. In *C. asiana,* sclerotised plates surrounding the gonopore are simple and square-like plates, while those of *C. tainanus* have forefinger and thumb-like projections that partially encircle the gonopore opening as seen in the Orientalis complex [42].

### Subgenus unplaced, Ornatus species group

#### *Culicoides palawanensis* Delfinado, 1961

Non-type material examined. ROK, Haenam, 34°38′15″N 126°19′55″E 23.06.16 (16 females), accession number PV111013–PV111017; 23.08.25 (one female), accession number PV111011; 23.09.27 (two females), accession number PV111012.

Diagnosis: According to Li *et al.* [36], the only species in the Ornatus group with a suite of characters including hairy eyes, SCo distributed on the flagellar segments 1–12, and spermathecae with a neck and lacking a sclerotised ring.

**Figure 6:**
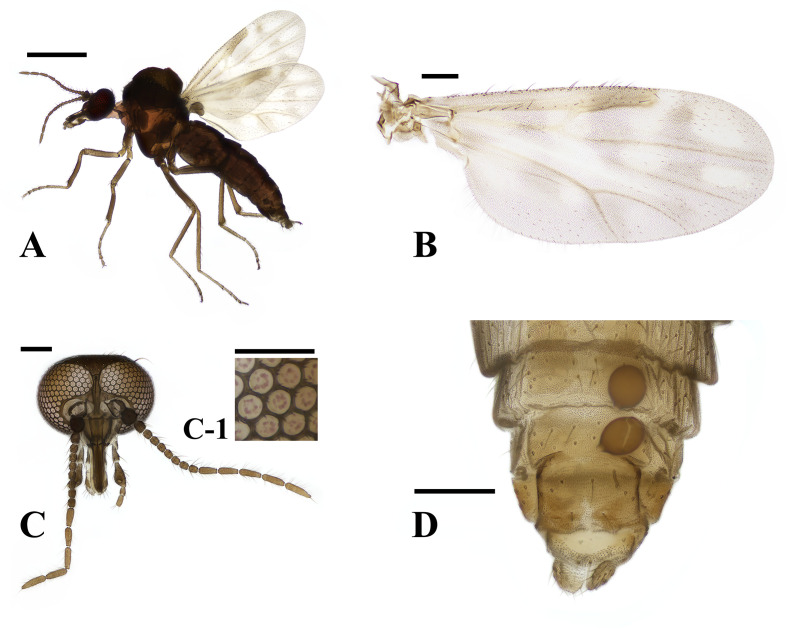
*Culicoides palawanensis* female (A) lateral view, (B) wing, (C) head, (D) abdomen. Scale bars = 0.5 mm [A]; 0.1 mm [B, C, and D]; 0.05 mm [C-1]).

Female (Figs. 2P and 6).

Body length 2.03 (1.47–2.45, *n* = 19) mm. Head: Eyes separated by 18 (17–19, *n* = 8) μm, or about 1.2 facets; hairy eyes, with three short pubescence located on each hexagonal edge of the ommatidium. Antennae with SCo on segments 1–12. AR 1.51 (1.38–1.58, *n* = 5); third palpal segment swollen distally, moderately deep, round sensory pit present. PR 2.31 (2.07–2.47, *n* = 6). P/H 0.72 (0.64–0.81, *n* = 8). Wings: 1.21 (0.89–1.33, *n* = 18) mm in length, 0.48 (0.43–0.61) mm in width, CR 0.65 (0.63–0.67). Wing pattern matches that of previous records, as seen in Figure 2P. Abdomen: Two spermathecae, oval-shaped and subequal in size, with developed necks; rudimentary spermatheca present; sclerotised ring absent. The larger spermatheca 59 (55–68, *n* = 8) × 50 (46–66) μm, smaller one 55 (52–57) × 49 (44–56) μm, and rudimentary spermatheca 20 (0.18–24) × 3 (3–4) μm.

Distribution: China, Thailand, Philippines, Indonesia, Malaysia, and the ROK.

Remarks: *C. palawanensis*’s wing pattern is similar to those of the domestic species *C. pallidulus* and *C. dendrophilus*. However, *C. pallidulu*s has larger pale spots on the wings and only one spermatheca, and *C. dendrophilus* has bare eyes.

Differences in body and wing length were observed between the individuals collected in June and those collected in August and September. The individuals collected in June had body lengths of 2.11 (1.83–2.25, *n* = 16) mm, wing lengths of 1.25 (1.18–1.33) mm, and wing widths of 0.56 (0.53–0.61) mm, while the individuals collected in August and September had body lengths of 1.57 (1.47–1.73, *n* = 3) mm, wing lengths of 0.99 (0.89–1.10) mm, and wing widths of 0.48 (0.43–0.54) mm. The *COI* sequences, however, did not differ between these individuals.

### Cytochrome c oxidase subunit 1 (*COI*) analysis

**Figure 7:**
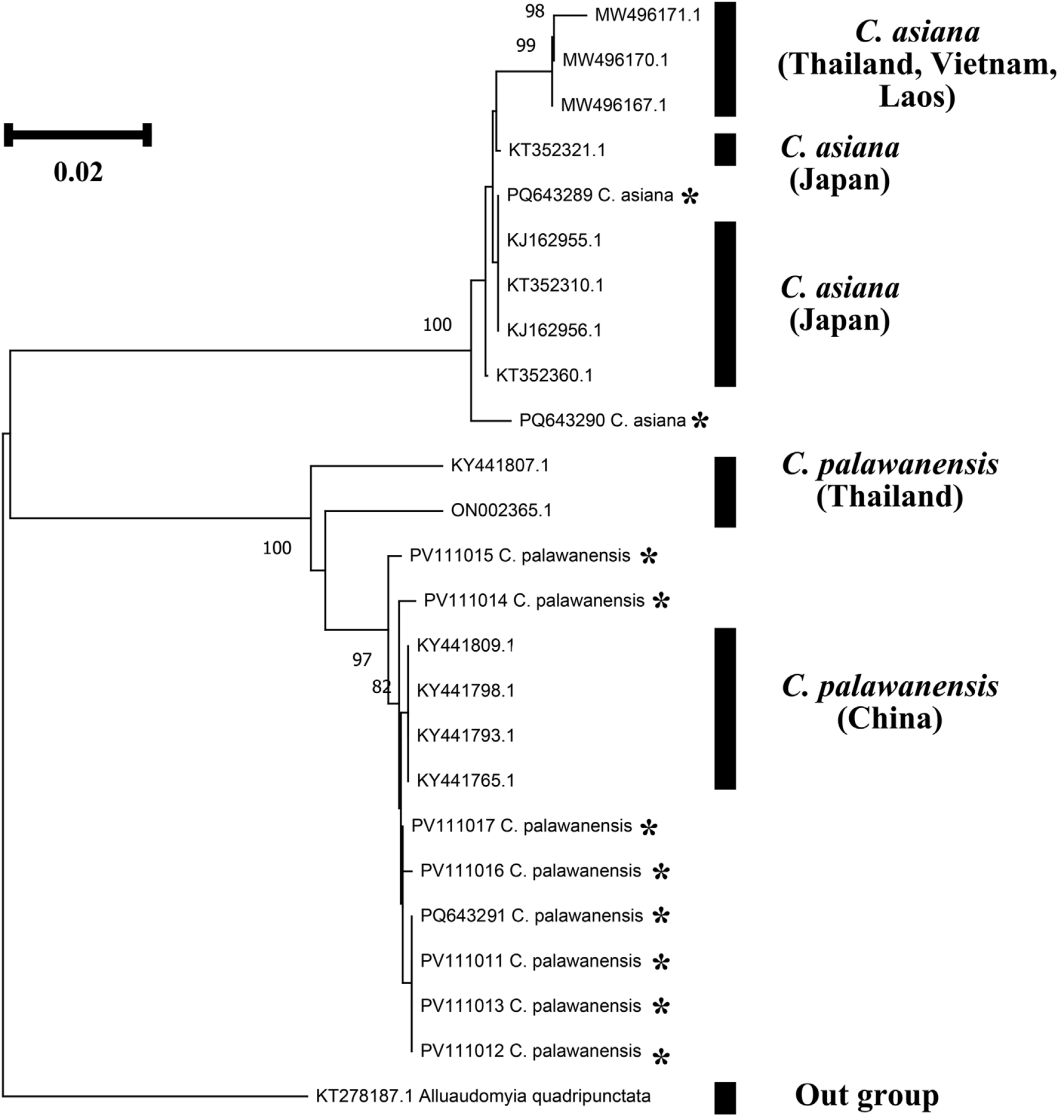
Phylogenetic trees featuring the *Culicoides* species previously unrecorded in the Republic of Korea, created using the neighbour-joining method based on *COI* sequences. Bootstrap values < 70 are not displayed. Sequences collected in this study are marked with an asterisk (*).

**Figure 8:**
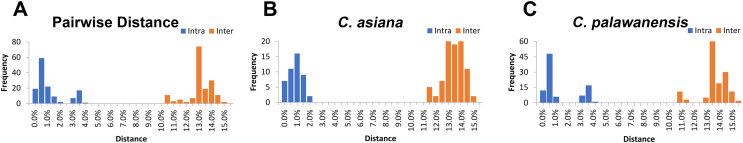
Genetic distance of newly recorded species: total pairwise distance (A), *C. asiana* (B), and *C. palawanensis* (C).

Phylogenetic analyses based on the *COI* region were performed to confirm the identification of the two newly recorded species based on the sequences of related species downloaded from GenBank (Table 7, Figs. 7 and 8). The *COI* sequences of *C. asiana* were divided into 2 clades in the phylogenetic tree, one representing populations from Japan and the ROK, and the other representing populations from Southeast Asia. The sequences obtained in the ROK demonstrated genetic distances of 0–0.98% when compared with those from Japan (KJ162955, KJ162956, KT352310, KT352321, and KT352360) and 0.98–1.84% when compared with those from Southeast Asia (MW496167, MW496170, and MW496171) (Fig. 8B).

**Table 7:** Pairwise distance (%) of newly recorded species: *C. asiana*, *C. palawanensis.*

	1	2	3	4	5	6	7	8	9	10	11	12	13	14	15	16	17	18	19	20	21	22	23	24	25
1. PQ643289.1 *C. asiana*	–																								
2. PQ643290.1 *C. asiana*	0.84	–																							
3. PQ643231.1 *C. palawanensis*	12.87	13.06	–																						
4. PV111011.1 *C. palawanensis*	12.87	13.06	0.00	–																					
5. PV111012.1 *C. palawanensis*	12.87	13.06	0.00	0.00	–																				
6. PV111013.1 *C. palawanensis*	12.87	13.06	0.00	0.00	0.00	–																			
7. PV111014.1 *C. palawanensis*	12.87	12.71	0.42	0.42	0.42	0.42	–																		
8. PV111015.1 *C. palawanensis*	12.67	12.87	0.56	0.56	0.56	0.56	0.70	–																	
9. PV111016.1 *C. palawanensis*	12.87	13.06	0.28	0.28	0.28	0.28	0.42	0.56	–																
10. PV111017.1 *C. palawanensis*	12.87	13.06	0.14	0.14	0.14	0.14	0.28	0.42	0.14	–															
11. KJ162955.1 *C. asiana*	0.00	0.84	12.87	12.87	12.87	12.87	12.87	12.67	12.87	12.87	–														
12. KJ162956.1 *C. asiana*	0.00	0.84	12.87	12.87	12.87	12.87	12.87	12.67	12.87	12.87	0.00	–													
13. KT352310.1 *C. asiana*	0.00	0.84	12.87	12.87	12.87	12.87	12.87	12.67	12.87	12.87	0.00	0.00	–												
14. KT352321.1 *C. asiana*	0.14	0.98	12.87	12.87	12.87	12.87	12.87	12.67	12.87	12.87	0.14	0.14	0.14	–											
15. KT352360.1 *C. asiana*	0.14	0.98	12.69	12.69	12.69	12.69	12.69	12.49	12.69	12.69	0.14	0.14	0.14	0.28	–										
16. MW496167.1 *C. asiana*	0.98	1.41	13.62	13.62	13.62	13.62	13.62	13.42	13.62	13.62	0.98	0.98	0.98	0.84	1.13	–									
17. MW496171.1 *C. asiana*	1.41	1.84	14.18	14.18	14.18	14.18	14.18	13.98	14.18	14.18	1.41	1.41	1.41	1.27	1.56	0.42	–								
18. MW496170.1 *C. asiana*	0.98	1.41	13.62	13.62	13.62	13.62	13.62	13.42	13.62	13.62	0.98	0.98	0.98	0.84	1.13	0.00	0.42	–							
19. KY441765.1 *C. palawanensis*	12.67	12.87	0.28	0.28	0.28	0.28	0.42	0.42	0.28	0.14	12.67	12.67	12.67	12.67	12.49	13.42	13.98	13.42	–						
20. KY441793.1 *C. palawanensis*	12.67	12.87	0.28	0.28	0.28	0.28	0.42	0.42	0.28	0.14	12.67	12.67	12.67	12.67	12.49	13.42	13.98	13.42	0.00	–					
21. KY441798.1 *C. palawanensis*	12.67	12.87	0.28	0.28	0.28	0.28	0.42	0.42	0.28	0.14	12.67	12.67	12.67	12.67	12.49	13.42	13.98	13.42	0.00	0.00	–				
22. KY441807.1 *C. palawanensis*	13.62	13.46	3.45	3.45	3.45	3.45	3.30	3.16	3.45	3.30	13.62	13.62	13.62	13.62	13.44	14.02	14.59	14.02	3.30	3.30	3.30	–			
23. KY441809.1 *C. palawanensis*	12.67	12.87	0.28	0.28	0.28	0.28	0.42	0.42	0.28	0.14	12.67	12.67	12.67	12.67	12.49	13.42	13.98	13.42	0.00	0.00	0.00	3.30	–		
24. ON002365.1 *C. palawanensis*	13.64	13.84	3.01	3.01	3.01	3.01	2.86	2.72	3.01	2.86	13.64	13.64	13.64	13.64	13.46	14.40	14.97	14.40	2.87	2.87	2.87	3.60	2.87	–	
25. KT278187.1 *A. quadripunctata* as outgroup	11.33	11.87	10.34	10.34	10.34	10.34	10.17	10.36	10.51	10.34	11.33	11.33	11.33	11.51	11.16	12.24	12.79	12.24	10.16	10.16	10.16	10.51	10.16	10.67	–

The *COI* sequences of *C. palawanensis* were divided into two clades, one representing collections from China and the other collections from Thailand. The newly obtained sequences from the ROK exhibited genetic distances of 0–0.70% when compared with the sequences from China (KY441765, KY441793, KY441798, and KY441809); however, they exhibited distances of 2.72–3.45% with the sequences recorded from Thailand (KY441807 and ON002365) (Fig. 8C).

## Discussion

Ecological surveys elucidating the seasonality, geographical distributions, and host preferences of *Culicoides* species are essential for identifying potential disease hotspots and for assessing the vector potential of individual species. In this study, we investigated the distribution of *Culicoides* at 15 locations throughout the southern part of the ROK at regular intervals, identifying 14 previously recorded species and 2 newly recorded species.

Previous research has shown that *C. arakawae* or *C. punctatus* were dominant nationwide [14, 26, 30, 65] exception on Jeju Island, while *C. tainanus* [28], *C. circumscriptu*s [30], *C. nipponensis* [14, 25, 27], and *C. erairai* [27] exhibited dominance in specific studies. In the current study, *C. arakawae* or *C. punctatus* were dominant on the mainland, while *C. matsuzawai* was dominant on Jeju Island (Tables 4 and 5).

We collected *C. arakawae* throughout the survey period, with the highest abundances seen in July and August in this study. This is consistent with previous studies reporting high abundances from June to August [14] and July to August [26, 61], but Kim *et al*. [27] reported high numbers of *C. arakawae* in September to October. For *C. punctatus,* abundance was high in June and July in the current study, and Cho and Chong [14] also reported high abundances in June and July. However, occurrences of the species were concentrated later in the year in several recent studies, in which high populations were seen in July to August [26, 30] or October [27]. *Culicoides tainanus* populations were highest from May to July in our study, they were also high in Cho and Chong [14] and Kim *et al.* [28], but Kim *et al.* [26] reported high abundance in August.

Another species, *C. nipponensis* was highly abundant in Gangjin, in late July (37.14%, 283) and in both collections in September (19.16%, 146; 28.08%, 214) (Tables 3 and 4). This pattern was similar to the high relative abundances noted from June to August by Cho and Chong [14] and in August by Kim *et al.* [30]. However, subsequent research by Kim *et al.* [28] identified a new domestic species *C. lungchiensis*, that morphologically resembles *C. nipponensis,* and suggested that *C. lungchiensis* may have been misidentified as *C. nipponensis* on Jeju Island. In the current study, *C. nipponensis* was only collected in Gangjin, and all specimens from Jeju Island that resembled *C. nipponensis* were identified as *C. lungchiensis*, suggesting that *C. nipponensis* may indeed not inhabit Jeju Island and the distribution record of *C. nipponensis* in the mainland should be reconsidered.

We recorded significant numbers of *C. matsuzawai* in July, and the species was highly dominant in goat sheds on Jeju Island (Tables 3 and 4). This temporal pattern contrasts with the those seen in previous studies, which reported low numbers of specimens from July to September [14] in Gyeonggi Province or from June to September [28] on Jeju Island, respectively. In the current study, this species emerged as a dominant presence on Jeju Island, and a considerable number were also collected in Jinju and Sacheon (Table 4). The COI sequences of several *C. matsuzawai* specimens from Jinju, Sacheon, and Jeju Island (PQ623695–704, PQ263828, PQ263829, PQ489391) were registered in GenBank [23] and coincided with other sequences in the database. Therefore, further investigation is required to understand why we encountered unusually large numbers of *C. matsuzawai*.

The composition of collected *Culicoides* populations appeared to vary depending on farm types and environmental conditions. Both *C. arakawae* (34.8%) and *C. punctatus* (39.1%) were dominant in the collection from cattle farms on the mainland, whereas *C. arakawae* (93.9%) exhibited the highest abundance on mainland goat farms. On Jeju Island, *C. tainanus* (32.24%) and *C. lungchiensis* (47.07%) were the dominant species on cattle farms, while *C. matsuzawai* (87.56%) were dominant on goat farms (Table 5). This finding reflects a significant environmental divergence between Jeju Island and the mainland. Regardless of farm types, the composition and relative abundances of *Culicoides* species varied according to the collection site and environment (Table 4, Fig. 3). This indicates that *Culicoides* populations may be influenced by numerous unknown factors. Therefore, future studies accounting for multiple environmental conditions, such as temperature, humidity, and the local habitat surrounding collection sites, will be essential for a comprehensive analysis of species distributions.

This study reported two species previously unrecorded in the ROK. *Culicoides asiana* was collected only on Jeju Island in August and September. The presence of this species has been confirmed in neighbouring countries, including Japan and China, as well as regions of Southeast Asia [6, 19]. Given the specimens collection location and phylogenetic placement (Table 4, Figs. 7 and 8B), the populations of *C. asiana* on Jeju Island appear to be more closely related to species in Japan than to those found elsewhere, which is not surprising given the geographical proximity of Japan to southern Korea. Additionally, the morphological similarities between *C. asiana* and *C. tainanus* suggest that *C. asiana* may have been overlooked in prior studies and may indeed be more widespread in Korea.

*Culicoides palawanensis* was collected in Haenam in June, August, and September. This species has been reported in southern China, Thailand, the Philippines, and Indonesia [18, 36, 61], so our records from Korea represent a significant northerly extension of the range of this species. Among publicly available *COI* sequences, the *C. palawanensis* sequences from Haenam exhibited greater similarity to sequences obtained from populations in Hainan, China (Figs. 7 and 8C). Considering this, along with the collection locations in China and the ROK, it is likely that *C. palawanensis* is more widespread in China than currently believed. Additionally, the notable variations in body size observed between specimens collected during different collection periods indicate the possibility of seasonal phenotypic variation, as has been observed in other species [39].

The current data are insufficient to determine whether the two species are native to the ROK or were introduced from external sources. Therefore, further research, for example, monitoring *Culicoides* along with seasonal airflow changes, is warranted to investigate introduction routes. Furthermore, these two species are primarily reported in tropical to subtropical climates [6, 61]. This suggests that, due to climate change, the ROK is experiencing influxes of new subtropical species from abroad. Thus, thorough epidemiological investigations, for instance analysing vector distributions and identifying potential disease candidates, are imperative to prevent and control disease transmission via *Culicoides* vectors.

This study identified six recorded species and one previously unrecorded species that have been identified as confirmed or potential disease transmission vectors in the ROK. Specifically, *C. arakawae*, *C. punctatus*, *C. oxystoma*, and *C. tainanus* have been shown to carry viruses domestically [47, 65], while *C. jacobsoni* [20] and *C. lungchiensis* [63] are potential vectors in the country. Additionally, *C. asiana* is a newly recorded species known to be capable of transmitting AKAV [6] and may transmit BTV (like *C. brevitarsis*) [6, 64]. Consequently, these seven species warrant more attention than the others. However, as pathogen isolation experiments were not performed in this study, leaving the current status of pathogens in these species and the identification of risk areas undetermined, further investigation is needed.

To implement effective control measures, it is necessary to accurately identify both the hosts and pathogens associated with *Culicoides*. There is, however, little information on the host range of Korean species of *Culicoides*. Among the 16 species collected in this study, firm host records exist for only six species. *Culicoides homotomus* and *C. nipponensis* are known to feed on cattle and chickens [24], while *C. kibunensis* and *C. punctatus* exhibit broader host ranges, targeting multiple mammal and bird species, although *C. punctatus* is known to prefer cattle and horses [40, 46, 58]. *Culicoides oxystoma* is known to feed on cattle, goats, sheep, and pigs, with a preference for cattle [24, 25, 37]. These previous studies help explain the species distribution seen in this study; species were collected in proximity to their respective hosts. Notably, *C. arakawae* is known to feed on cattle, chickens, goats, and pigs [24, 37], and is thought to prefer avian species [66]. However, it has consistently shown high abundances around mammal farms in the ROK [26, 30, 65]. In the current survey, *C. arakawae* exhibited high collection rates on both cattle and goat farms, being consistently abundant at all the tested goat sheds on the mainland. Thus, it seems possible that host preference of *C. arakawae* is not perfectly understood. However, there are limitations in the current study: the influence of nearby wild bird or poultry populations, the dispersal ability of *Culicoides* species, and larval habitat were not measured. Further research, such as direct blood analyses from engorged *Culicoides* and the measurement of host-specific blood-feeding rates, will therefore be necessary to achieve a comprehensive understanding of host preferences.

In conclusion, this study provided updated information about the distributions of *Culicoides* species in the southern region of the ROK, identifying two previously unrecorded species, *C. asiana* and *C. palawanensis*. As a comprehensive survey on goat farms has not been conducted to date, the data obtained from this survey are expected to serve as a valuable foundational resource for the monitoring and analysis of *Culicoides* vectors associated with livestock diseases in this country. Until now, only four species have been directly associated with viruses within the ROK. Thus, the identification of a new potential disease vector, *C. asiana*, raises concerns regarding the introduction of new diseases and disease vectors from abroad. Therefore, the ongoing monitoring of *Culicoides* vectors and potential foreign incursions will be essential for ensuring the health and hygiene of domestic livestock in the future.
